# Parabolic relationship between *SMAD3* expression level and the reprogramming efficiency of goat induced mammary epithelial cells

**DOI:** 10.3389/fcell.2022.1002874

**Published:** 2022-10-14

**Authors:** Yulian Wu, Dandan Zhang, Sheng Ye, Quanhui Liu, Ben Huang

**Affiliations:** ^1^ Guangxi Academy of Medical Sciences and the People’s Hospital of Guangxi Zhuang Autonomous Region, Nanning, China; ^2^ State Key Laboratory for Conservation and Utilization of Subtropical Agro-8 Bioresources, Guangxi University, Nanning, China; ^3^ School of Animal Science and Technology, Guangxi University, Nanning, China

**Keywords:** somatic cell reprogramming, Smad3, induced mammary epithelial cells, cell fate determination, Tet On

## Abstract

Mammary epithelial cells are the only cells of mammary glands with lactation capacity. They are closely related to mammary development and milk yield. Our earlier studies showed that the transformation of goat fibroblasts into induced mammary epithelial cells (iMECs) was closely correlated with *SMAD3* overexpression. Therefore, we further explored the role of *SMAD3* on iMECs reprogramming in this study. The *SMAD3* gene was overexpressed in goat ear fibroblasts using the tetracycline-induced expression method. The outcomes demonstrated that goat ear fibroblasts can be converted into iMECs by overexpressing the *SMAD3* gene. In contrast, it was discovered that *SMAD3* downregulation by RNA interference significantly decrease the reprogramming efficiency of iMECs. These results show that *SMAD3* plays a key regulatory role in the reprogramming of iMECs. Surprisingly, we also found a parabolic relationship between *SMAD3* expression level and iMECs reprogramming efficiency, and that the reprogramming efficiency was maximum when the addition of doxycycline concentration was 5 μg/ml. In light of this, our findings may offer new perspectives on the regulatory mechanism governing mammary epithelial cell fate in goats as well as a fresh approach to studying mammary development and differentiation *in vitro*.

## Introduction

The development of multicellular organisms is a delicate and complex process, which is dynamically regulated to maintain long-term coordination and stability in the body. Different cell groups have their unique steady state and undergo cell differentiation along established developmental trajectories. Cells hardly ever alter their course and develop into other cell types in the natural condition. An earlier investigation shown that overexpression the transcription factor MyoD can reprogram mouse embryonic fibroblasts into myoblasts (Davis et al., 1987). The conventional notion of cell differentiation was put to the test by this study, which showed that cells have plasticity and that their identity may be modified. The gene expression profile of somatic cells can be modified by external factors induction, and subsequently, somatic cells have stimulated the potential to achieve other cell fates bypassing the pluripotent cell stage. This phenomenon is known as lineage reprogramming or transdifferentiation, which can be regulated by signaling pathways, epigenetic modifications, and cellular metabolic processes (Bannister and Kouzarides, 2011; Yoo et al., 2011; Li et al., 2013; Iwafuchi-Doi and Zaret, 2014; Soufi et al., 2015).

In recent years, transdifferentiation has been achieved by adding particular transcription factors or miRNAs into a variety of cells, including cardiomyocytes (Ieda et al., 2010; Wada et al., 2013), hepatocytes (Huang et al., 2014; Zhu et al., 2014), neural stem cells (Sheng et al., 2012) and neurons (Kim et al., 2011; Wang et al., 2013), astrocytes (Caiazzo et al., 2015), endothelial cells (Han et al., 2014), pancreatic β cells (Li et al., 2014), hematopoietic stem cells (Batta et al., 2014), and macrophages (Bussmann et al., 2009). As a non-viral, non-integrating form, small molecule compounds have the unique advantages of rapid cell entry through high permeability, relatively clear targets, and relatively controlled effects, showing great potential in transdifferentiation studies. To date, various studies have reported the use of small molecule compounds alone for transdifferentiation to generate neural stem cells (Han et al., 2016; Zheng et al., 2016), neurons (Cheng et al., 2015; Li et al., 2015; Wenxiang et al., 2015), endothelial cells (Wang et al., 2016), cardiomyocytes (Fu et al., 2015) and hepatocytes (Wang et al., 2016).

Currently, we have identified the combination of five small molecule compounds (1 μM TTNPB (B), 10 μM Forskolin (F), 10 μM RepSox (R), 10 μM Tranylcypromine (T) and 500 μg/ml VPA (V), BFRTV), which can induce reprogramming in goat ear fibroblasts (GEFs) to give rise to chemically induced MECs (CiMECs) (Zhang et al., 2021). The molecular mechanisms behind the growth, development, and lactation of the mammary gland can be studied using goat mammary epithelial cells. High-quality iMECs were generated by small molecule compounds induction and serve as a cellular platform to study mammary gland-related biological mechanisms *in vitro*. We next determined that the essential small molecule for this reprogramming was RepSox (R), an inhibitor of TGF receptor 1 (TGFβR1). Our previous findings also indicated that overexpression of *SMAD3*, one of the TGFβR1 downstream genes, may be crucial for the generation of induced mammary epithelial cells (iMECs). As a key transcription factor mediating TGFβ signaling, SMAD3 plays a key role in a variety of biological processes and performs multiple functions, including growth arrest, differentiation, apoptosis, and epithelial-mesenchymal transition (EMT) (Millet and Zhang, 2007; Padua and Massagué, 2009; Ikushima and Miyazono, 2010; Massagué, 2012). SMAD3 is involved in the development and differentiation of mammary epithelial cells (Yang et al., 2002; Yoo et al., 2009), but the precise mechanism of SMAD3-mediated reprogramming have not been thoroughly explored. Therefore, in the present study, we further elucidated the function of SMAD3 in the reprogramming of iMECs and investigated the relationship between *SMAD3* expression level and reprogramming efficiency, complementing the reprogramming mechanism of iMECs in goats while providing insights into mammary epithelial cell fate determination.

## Materials and methods

### Plasmids and cells

The pLVX-TetOne-Puro, psPAX2, and pMD2.G plasmids were purchased from Hunan Fenghui Biotechnology Co. The pUC57 2As, pLVX-IRES-ZsGreen1, and pSicoR-Ef1a-mCherry plasmids were pre-constructed and preserved by the School of Animal Science and Technology, Guangxi University. HEK-293T cells were preserved by the School of Animal Science and Technology, Guangxi University.

### Cell culture and induction medium

Goat ear fibroblasts (GEFs) were cultured in high glucose DMEM (GIBCO) supplemented with 10% fetal bovine serum (FBS, GIBCO) (Fibroblasts culture medium, FM).

BFTV induction medium contained Neurobasal Medium (GIBCO), KnockOut DMEM-F12 (GIBCO), KSR (GIBCO), 100 × N2 (GIBCO), 50 × B27 (GIBCO) supplements, 1% GlutaMAX (GIBCO), we refer to it as N2B27-based medium; and supplemented with four small molecule cocktail, 1 μM TTNPB (B), 10 μM Forskolin (F), 10 μM Tranylcypromine (T) and 500 μg/ml VPA (V); R induction medium was supplemented with 10 μM RepSox(R) in N2B27-basal medium.

### Plasmid construction

The goat SMAD family member 3 (SMAD3) mRNA sequence (GenBank Accession No. XM_013966776.2), pUC57 2As, and pLVX-IRES-ZsGreen1 plasmids were used to design the primers listed in Table 1. *SMAD3*, P2A, and ZsGreen1 fragments carrying homologous arms were amplified by RT-PCR and spliced into SMAD3-P2A-ZsGreen1 by overlap extension PCR (SOE PCR). SMAD3-P2A-ZsGreen1 inserted into the *EcoRI/BamHI* sites of pLVX-TetOne-Puro (pLVX; empty vector). Then, the doxycycline (Dox)-induced overexpression of *SMAD3* lentiviral plasmid (pLVX-TetOne-SMAD3-P2A-ZsGreen1-Puro) was constructed. Construction of the lentiviral plasmid was confirmed by bacterial liquid PCR, double enzyme digestion, and sequencing. (See Supplementary Material for details of this part of the reaction system and procedures).

**TABLE 1:** Primers for vector construction and identification.

Primer name	Primer sequence(5′–3′)
G-SMAD3-CF	CCT​ACC​CTC​GTA​AAG​AAT​TCG​CCA​CCA​TGT​CGT​CCA​TCC​TGC​CTT​TCA​CT
G-SMAD3-CR	GAA​GTT​CGT​GGC​AGA​CAC​GCT​GGA​GCA​GCG​G
P2A-CF	CTC​CAG​CGT​GTC​TGC​CAC​GAA​CTT​CTC​TCT​GTT​AAA​GCA​AGC
P2A-CR	GCT​TGG​ACT​GGG​CCA​TAG​GCC​CGG​GGT​TTT​CTT​CAA​CAT​C
ZsGreen1-CF	GAA​AAC​CCC​GGG​CCT​ATG​GCC​CAG​TCC​AAG​CAC​GG
ZsGreen1-CR	CAG​GGG​AGG​TGG​TCT​GGA​TCC​TCA​GGG​CAA​GGC​GGA​GCC​G
shRNA1-SMAD3-F	AAC​GCC​TCA​GTG​ACA​GCG​CCA​TCT​TTC​AAG​AGA​AGA​TGG​CGC​TGT​CAC​TGA​GGC​TTT​TTT​C
shRNA1-SMAD3-R	TCG​AGA​AAA​AAG​CCT​CAG​TGA​CAG​CGC​CAT​CTT​CTC​TTG​AAA​GAT​GGC​GCT​GTC​ACT​GAG​GCG​TT
shRNA2-SMAD3-F	AAC​CAT​CCG​CAT​GAG​CTT​CGT​CAA​TTC​AAG​AGA​TTG​ACG​AAG​CTC​ATG​CGG​ATG​TTT​TTT​C
shRNA2-SMAD3-R	TCG​AGA​AAA​AAC​ATC​CGC​ATG​AGC​TTC​GTC​AAT​CTC​TTG​AAT​TGA​CGA​AGC​TCA​TGC​GGA​TGG​TT
shRNA3-SMAD3-F	AAC​GGA​TTG​AGC​TGC​ACC​TGA​ACG​TTC​AAG​AGA​CGT​TCA​GGT​GCA​GCT​CAA​TCC​TTT​TTT​C
shRNA3-SMAD3-R	TCG​AGA​AAA​AAG​GAT​TGA​GCT​GCA​CCT​GAA​CGT​CTC​TTG​AAC​GTT​CAG​GTG​CAG​CTC​AAT​CCG​TT
shRNA-NC-F	AAC​CCT​AAG​GTT​AAG​TCG​CCC​TCG​TTC​AAG​AGA​CGA​GGG​CGA​CTT​AAC​CTT​AGG​TTT​TTT​C
shRNA-NC-R	TCG​AGA​AAA​AAC​CTA​AGG​TTA​AGT​CGC​CCT​CGT​CTC​TTG​AAC​GAG​GGC​GAC​TTA​ACC​TTA​GGG​TT
pLVX-TetOn-F	GCT​TTG​CTT​ATG​TAA​ACC​AG
pLVX-TetOn-R	CTT​AGG​TTG​GAG​TGA​TAC​AT
pSicoR-F	CACAAAAGGAAACTCACC
pSicoR-R	GCCAGTACACGACATCAC

Three interference targets were designed for the mRNA sequence of goat *SMAD3*, as listed in Table 1. The oligo DNA was annealed to synthesize shRNA1/2/3-SMAD3 and the nonsense shRNA (NC; negative control, a disrupted and meaningless sequence was inserted into the vector to exclude inserted sequence from potentially interfering with the experiment results). The goat *SMAD3* gene lentiviral interference vector (pSicoR-Ef1a-mCherry-shRNA1/2/3-SMAD3) and its NC control vector (pSicoR-Ef1a-mCherry-shRNA1/2/3-SMAD3) were constructed by cleaving the pSicoR-Ef1a-mCherry vector (pSicoR; empty vector) with *HpaI* and *Xhol* enzymes, ligating the shRNA to linearized vector using T4 DNA ligase. Bacterial liquid PCR and sequencing provided proof that the lentiviral interference plasmids had been successfully created. The oligo DNA fragments were synthesized by Sangon Biotech, China.

### Isolation and culture of goat ear fibroblasts

Goat’s ears were disinfected and dehaired, and small pieces of ear margin tissue were dissected. The tissues were washed once with 75% (v/v) alcohol and then three times with PBS (containing 2% penicillin-streptomycin). The tissues were then cut into 1-mm^3^ sections and plated in a 100-mm dish. The dish was placed in a carbon dioxide (CO_2_) incubator (37°C, 5% CO_2_). When the tissue mass reached translucence, FM was added. Once the confluence of GEF reached 80%–90%, it was passaged.

### Screening of suitable concentration of puromycin

The GEFs were cultured in 24-well plates at a density of 1 × 10^4^ cells/well in FM and washed with PBS after 48 h, replace with the new culture medium. Different concentrations of puromycin (1, 2, 3, 4, 5 μg/ml) were added to the cell culture medium, three replicate wells were treated with each concentration and changed daily to a fresh cell culture medium containing the corresponding concentration of puromycin.

### Lentivirus infection

Lentiviral recombinant plasmid/vector (2.5 μg) was cotransfected together with the pMD2.G (1 μg) and psPAX2 (1.5 μg) plasmids into HEK-293T cells using Lipofectamine 3,000 (Invitrogen) in 6-cm dishes and Dox was added to the culture medium. The viral supernatant was collected after transfection for 48–72 h at 37°C and 5% CO_2_. The supernatant was centrifuged (2,000 rpm for 10 min at 4°C), filtered through 0.45-μm filters, and then placed onto GEFs supplemented with 8 ng/μL polybrene (Solarbio) and Dox. Two days later, microscopic images, evaluated the efficiency of viral infection, and the infected cells were collected, counted, and plated onto dishes (2.5 × 10^5^ cells for a 6-cm dish) with FM addition of puromycin (3 μg/ml).

### Generation of SMAD3 overexpression-induced mammary epithelial cells (SMAD3-iMECs)

Dox-induced overexpression of SMAD3 cells (GEFs-SMAD3) seeded at a density of 5 × 10^4^ cells per well in a 4-well plate or at 5 × 10^5^ cells per 60-mm dish and cultured with fibroblasts culture medium in an incubator at 37°C in a humidified atmosphere of 5% CO_2_. After 8 h, the FM was replaced by BFTV, and Dox was added. The culture was continued for 8 days, and the induction medium was refreshed every 2 days. GEFs were induced by R induction medium to generate R-CiMECs acted as positive control cells.

### Real-time fluorescence quantitative PCR

Total RNA was isolated using TRIzol Reagent (Servicebio, G3013) and cDNA synthesis was performed using a HiScript^®^ III 1st Strand cDNA Synthesis Kit (+gDNA wiper) (Vazyme, R223-01). RT-qPCR was performed using ChamQ Universal SYBR^®^ qPCR Master Mix (Vazyme, Q711-02/03). The data were analyzed using the 2^−ΔΔCT^ method. The primer sequences for RT-qPCR in this study are listed in Table 2. The expression of genes was normalized to that of the internal control gene *GAPDH*, and the expression fold change for the samples was calculated relative to the expression of the fibroblasts (GEFs).

**TABLE 2:** Primers for RT-qPCR.

Gene name	Primer sequence(5′–3′)
*GATA3*	F: CAC​CCC​TCT​CTG​GCG​ACG​A
	R: ACA​GTT​TGC​ACA​GGA​CGT​ACC
*SMAD3*	F: AGG​AGA​AGT​GGT​GCG​AGA​AG
	R: ATCCAGGGACCTGGGGA
*GAPDH*	F: CGT​TGC​CAT​CAA​TGA​CCC​CTT
	R: CGT​ACT​CAG​CAC​CAG​CAT​CAC​C
*MSX2*	F: GAGGAACGCCGCGTCAA
	R: GTG​GGG​CTC​ATG​TGT​CTT​GG
*VIMENTIN*	F: ACC​GCT​TCG​CCA​ACT​ACA​TCG
	R: ACT​TGC​CCT​GTC​CCT​TGA​GC
*FBN1*	F: CCG​AGT​GTG​TGA​CGA​TGT​GA
	R: ATC​GCA​GGT​CTG​GTT​GTC​AG
*COL3A1*	F: ACT​TTT​CGC​TCT​GCT​TCA​TCC​C
	R: ACG​CAT​ATT​TGG​CAC​GGT​TC
*LTF*	F: AAG​AAC​CTC​AGG​GAA​ACC​GC
	R: TCC​ACT​GCT​GGC​ACT​TAC​TC
*KRT18*	F: CTC​CTG​CAC​CTG​GAG​TCA​GA
	R: CGC​CAA​GAC​TGA​AAT​CCT​CC

### Saturated oil red O staining

The cells were fixed with 4% paraformaldehyde for 10 min, and then the cells were stained for 15 min with oil red O staining solution (Solarbio, China, G1262). The cells were then rinsed with 60% isopropanol for 15–30 s and washed three times with sterile water. Mayer’s hematoxylin was added to stain the nuclei for 20 s, and the cells were washed with sterile water three times.

### Immunofluorescence staining

After fixation with 4% paraformaldehyde (Sigma) at room temperature for 30 min, the cells were permeabilized with 1% Triton X-100 and blocked with 10% donkey serum at 37°C for one and a half hours. Primary antibodies were incubated with the cells at the appropriate dilutions at 4°C overnight in blocking buffer. The next day, the cells were probed with respective secondary antibodies for 1 h in the dark at room temperature. Nuclei were stained with Hoechst 33342. Antibody details are listed in the Supplemental Material.

### Western blot

Cells were lysed in denaturing lysis buffer containing protease inhibitors (RIPA, Solarbio, Beijing, China) for 30 min on ice and centrifuged (12,000 rpm) for 10 min at 4°C. Protein concentrations in the lysates were determined by a BCA protein assay kit (Solarbio, Beijing, China). Approximately 30 μg of protein was separated by 12% SDS-PAGE and transferred to a nitrocellulose filter membrane that was blocked with 5% nonfat dried milk in Tris-buffered saline containing 0.05% Tween 20 (pH 7.6) for 1 h at 25°C. Subsequently, the membranes were incubated overnight at 4°C with primary antibodies. Then, the blots were incubated with secondary antibodies for 1 h at room temperature. After washing three times with TBST, immunostaining was visualized using Western blotting detection reagents (Bio-Rad, United States). Antibody details are listed in the Supplemental Material.

### Quantification and statistical analysis

Independent pavement-like cell colonies were formed on day 4 of induction, and this compact pavement-like cell colonies were defined as primary colonies. Reprogramming efficiency (cell colony formation rate) was determined by the percentage of the number of independent primary colonies to the number of seeded fibroblasts. Efficiency (%) = No. of primary colonies/No. of seeded cells × 100%.

Statistical analyses for differential gene expression (Figures 3C, 4C, 5A) and comparison of reprogramming efficiency (Figures 3B,E, 4B,E) were performed in GraphPad. Significance was calculated with Student’s t test or one-way ANOVA. Data are presented as mean ± SEM. *p < 0.05, **p < 0.01, ***p < 0.001.

## Results

### Overexpression and interference of *SMAD3* gene in goat fibroblasts

**FIGURE 1:**
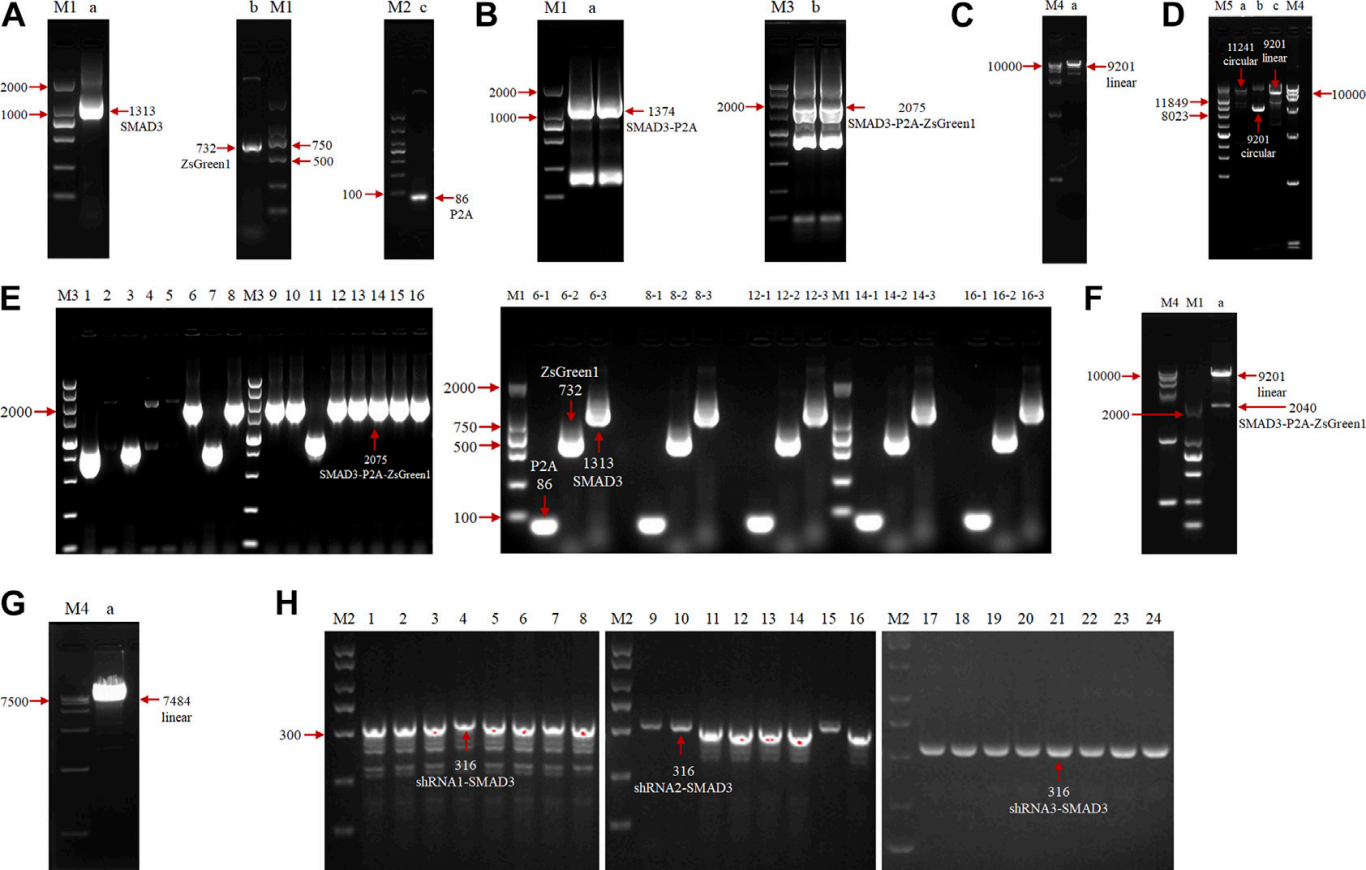
Agarose gel electrophoresis analysis of overexpression and construction of eukaryotic expression vectors for the interfering goat *SMAD3* gene. **(A)** PCR amplification of *SMAD3*, P2A, and ZsGreen1 sequences, respectively. M: marker (M1: DL2000, M2: DL1000); lane a: *SMAD3* gene, 1313 bp; lane b: ZsGreen1 sequence, 732 bp; lane c: P2A sequence, 86 bp. **(B)** SOE PCR ligation of *SMAD3*, P2A, and ZsGreen1 fragments. M: marker (M3: DL5000); lane a: SMAD3-P2A fragment, 1374 bp; lane b: SMAD3-P2A-ZsGreen1 fragment, 2075 bp **(C)** Enzymatic cleavage of the pLVX-TetOne-Puro vector. M: marker (M4: DL15000); lane a: linearized pLVX-TetOne-Puro vector, 9201 bp. **(D)** Homologous recombination reaction. M: marker (M5: Supercoiled DNA Ladder Marker); lane a: recombination product, 11241 bp; lane b: cyclic pLVX-TetOne-Puro vector, 9201 bp; lane c: linearized pLVX-TetOne-Puro vector, 9201 bp. **(E)** PCR identification of recombinant product transformed bacterial solution. Lanes 1–16: first bacterial fluid PCR bands of 16 monoclonal colonies, positive colony product (SMAD3-P2A-ZsGreen1) size 2075 bp; lanes 6/8/12/14/16–1/2/3: 6, 8, 12, 14, 16 positive colonies second bacterial fluid PCR bands, positive colonies all can produce 86 bp (P2A), 732 bp (ZsGreen1) and 1313 bp (*SMAD3*) bands, respectively. **(F)** Plasmid double digestion identification. Lane a: positive plasmids were digested by *EcoRI* and *BamHI* to become linearized pLVX-TetOne-Puro vector as well as SMAD3-P2A-ZsGreen1 bands (without homologous arms), size 9201 and 2040 bp, respectively. **(G)** Enzymatic cleavage of the pSicoR-Ef1a-mCherry vector. Lane a: linearized pSicoR-Ef1a-mCherry vector, 7484 bp. **(H)** PCR identification of ligated products transformed bacterial solution. Lanes 1–24: bacterial fluid PCR bands of 8 monoclonal colonies each of 3 interfering target sequences, positive colonies products all 316 bp in size (shRNA1/2/3-SMAD3).

To investigate the function of the goat *SMAD3* gene in the reprogramming process of induced mammary epithelial cells, we regulated the expression level of *SMAD3* gene in goat fibroblasts. A Dox-induced eukaryotic expression vector for goat *SMAD3* overexpression, pLVX-TetOne-SMAD3-Puro, was successfully created through a series of PCR experiments (Figures 1A–E). In this vector, the expression of the P2A-linked green fluorescent protein ZsGreen1 allowed us to estimate the expression of *SMAD3* in an approximate manner. We also obtained the eukaryotic expression vector pSicoR-Ef1a-mCherry-shRNA-SMAD3, which targets the knockdown of the *SMAD3* gene in goats (Figures 1F–H).

**FIGURE 2:**
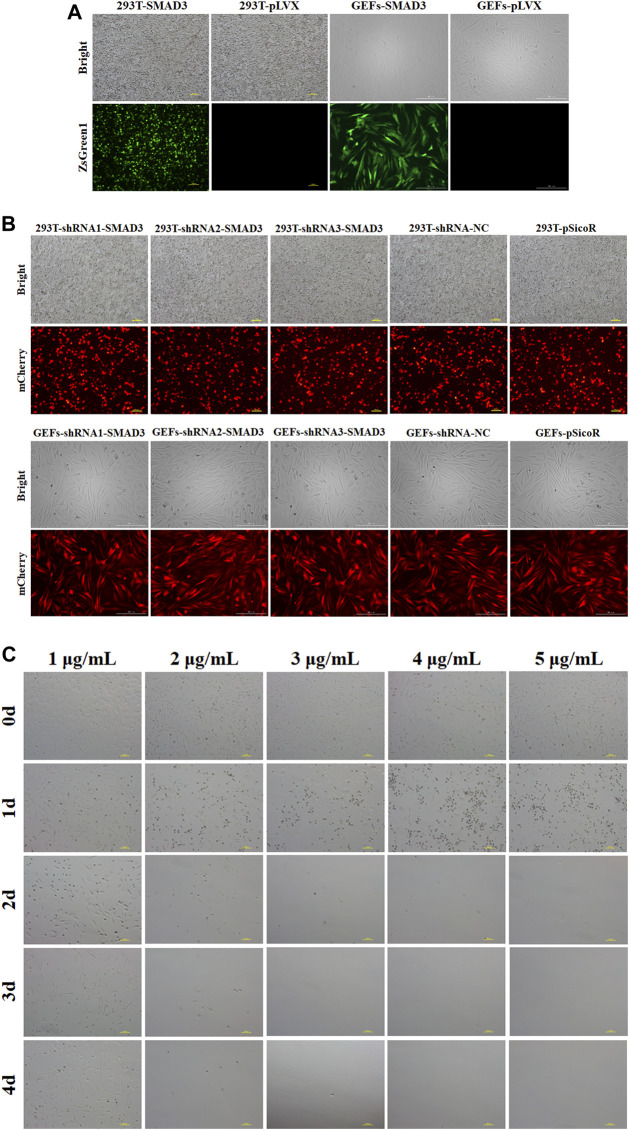
Stable overexpression and interference of goat *SMAD3* gene cell lines (GEFs-SMAD3 and GEFs-shRNA-SMAD3) were obtained. **(A)** Fluorescence rate of pLVX-TetOne-SMAD3-Puro packaged lentivirus and virus supernatant infected goat fibroblasts for 48 h. Yellow scale bar, 100 μm, white scale bar, 200 μm. **(B)** Fluorescence rate of pSicoR-Ef1a-mCherry-shRNA-SMAD3 packaged lentivirus and virus supernatant infected goat fibroblasts for 48 h. Yellow scale bar, 100 μm, white scale bar, 200 μm. **(C)** Screening of suitable concentrations of puromycin. Scale bar, 100 μm.

We selected the lentivirus-mediated method to infect GEFs after lentiviral packaging of the target plasmid in order to increase transfection efficiency and obtain cell lines with long-term stable overexpression and interference with the goat *SMAD3* gene. The results showed that the infection efficiency of pLVX-TetOne-SMAD3 lentivirus was close to 60% (Figure 2A), while the infection efficiency of pSicoR-Ef1a-mCherry-shRNA1/2/3-SMAD3 lentivirus virus could reach 80%–90% (Figure 2B). In addition, the outcomes of puromycin screening suggested that 3 μg/ml could be used as the ideal dose to utilize for cell selection (Figure 2C; Table 3).

**TABLE 3:** Days of fully causing death at different concentrations of puromycin.

Concentration of Puromycin(μg/mL)	1	2	3	4	5
Day of all of GEFs cells were killed	—	4	3	2	2

### The reprogramming efficiency of induced mammary epithelial cell tends to increase and then decrease with increasing *SMAD3* expression levels

**FIGURE 3:**
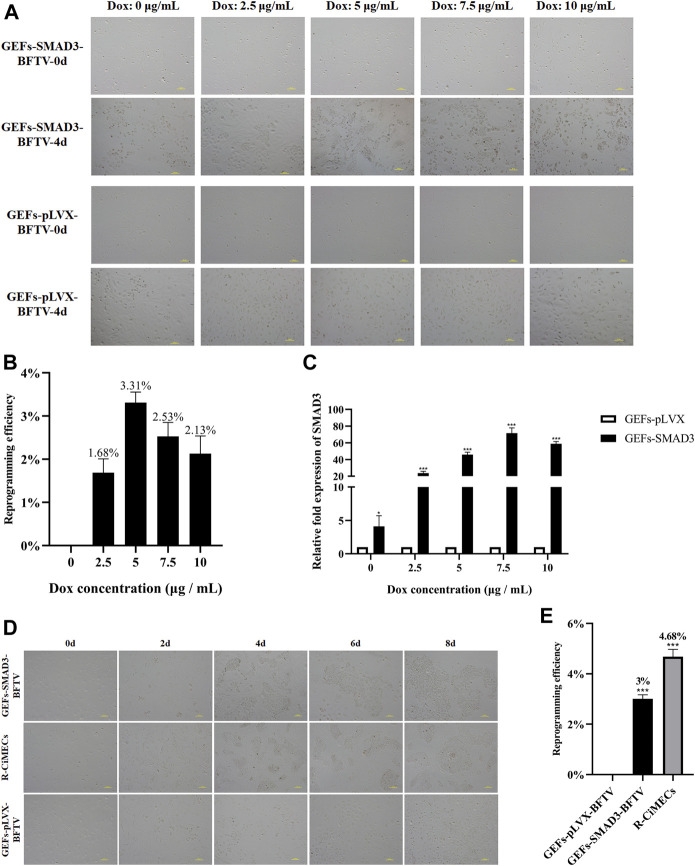
Effect of *SMAD3* expression on reprogramming efficiency of goat induced mammary epithelial cells. **(A)** The ability of cells to form independent clones within 4 days under different concentrations of Dox induction. Scale bar, 100 μm. **(B)** Reprogramming efficiency of goat induced mammary epithelial cells under different concentrations of Dox treatment (Efficiency (%) = No. of primary colonies/No. of seeded cells × 100%). **(C)** Changes in *SMAD3* gene expression levels in goat fibroblasts induced by different concentrations of Dox. (Due to the specificity of the vector, there was a small leakage of expression at Dox = 0 μg/ml) *n* = 3 biological replicates. Data are represented as the mean ± SEM. *p < 0.05, ***p < 0.001 (Student’s t test). **(D)** Morphological changes during overexpression of SMAD3-induced reprogramming of goat fibroblasts into mammary epithelial cells (Dox = 5 μg/ml). Scale bar, 100 μm. **(E)** Overexpression of *SMAD3* significantly improves the reprogramming efficiency of goat induced mammary epithelial cells (Dox = 5 μg/ml). Scale bar, 100 μm. *n* = 3 biological replicates. Data are represented as the mean ± SEM. ***p < 0.001 (one-way ANOVA).

Subsequently, we determined the effects of different concentrations of doxycycline (Dox) on inducing GEFs-SMAD3 to reprogram into SMAD3-iMECs. Under the addition of various Dox concentrations (2.5, 5, 7.5, and 10 g/ml), GEFs-SMAD3 allows incremental epithelization from the initial typical fibroblasts during the reprogramming process. Independent and compact epithelial cell-like colonies were formed on day 4 of the reprogramming process (Figure 3A), but the state and number of colonies varied depending on the Dox concentration groups. At a Dox concentration of 5 g/ml, the reprogramming efficiency was 3.31%, which was the highest among the various groups (Figure 3B). However, GEFs-pLVX cells as the empty control group failed to form any independent and compact epithelial cell colonies under the same culture conditions. The results also showed that the expression levels of *SMAD3* increased when the concentration of Dox increased, peaked at a concentration of 7.5 μg/ml but decreased at a concentration of 10 μg/ml (Figure 3C). The aforementioned findings thus indicated that there was a parabolic rather than linear relationship between the reprogramming efficiency of iMECs and the expression levels of *SMAD3*. Therefore, we decided 5 g/ml was the optimal concentration of Dox for subsequent induction assays. As induction time increased, GEFs-SMAD3 underwent epithelialization and formed small epithelial cell-like aggregates; followed by the independent paver-like clones formed, and the clonal area gradually increased and eventually stabilized (Figure 3D). Compared to the GEFs-pLVX, the reprogramming efficiency of GEFs-SMAD3 was greatly enhanced, but slightly lower than the positive control R-CiMECs (3% vs. 4.68%) (Figure 3E).

### The *SMAD3* gene plays a key role in the acquisition of induced mammary epithelial cells in goats

**FIGURE 4:**
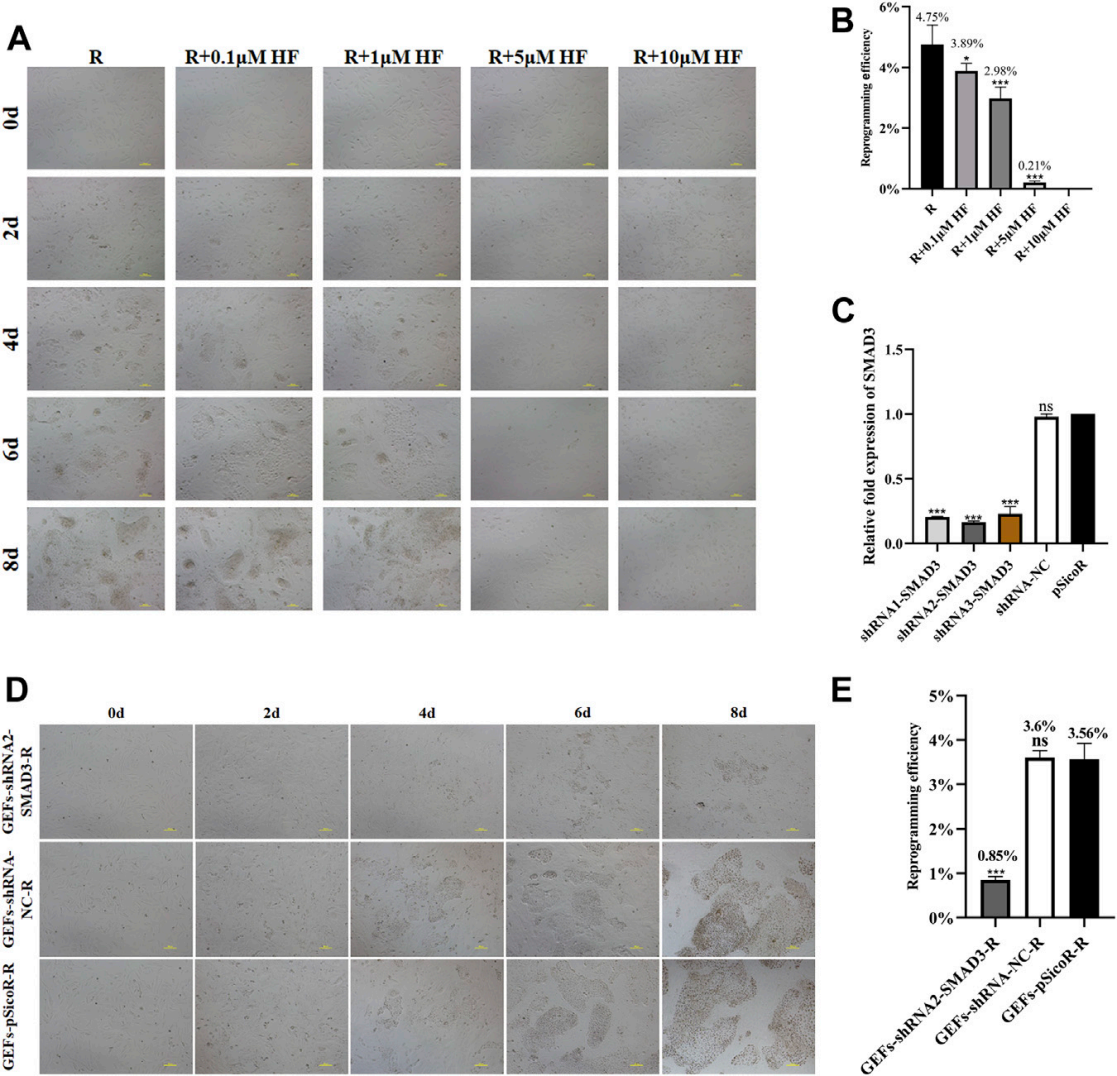
*SMAD3* downregulation impedes the reprogramming process of induced mammary epithelial cells in goats. **(A)** Effect of different concentrations of HF on the reprogramming process of induced mammary epithelial cells in goats. Scale bar, 100 μm. **(B)** Inhibitory effect of different concentrations of HF on the reprogramming efficiency of induced mammary epithelial cells in goats. *n* = 3 biological replicates. Data are represented as the mean ± SEM. *p < 0.05, ***p < 0.001 (one-way ANOVA). **(C)** Expression levels of *SMAD3* gene in three interfering target sequences in the corresponding cell lines (GEFs-shRNA1/2/3-SMAD3). *n* = 3 biological replicates. Data are represented as the mean ± SEM. ***p < 0.001 (one-way ANOVA). **(D)** Effect of interfering with *SMAD3* expression on the reprogramming process of induced mammary epithelial cells in goats. Scale bar, 100 μm. **(E)** Interfering with *SMAD3* expression significantly reduces the reprogramming efficiency of induced mammary epithelial cells in goats. Scale bar, 100 μm; *n* = 3 biological replicates. Data are represented as the mean ± SEM. ***p < 0.001 (one-way ANOVA).

We found that addition of SMAD3 inhibitor Halofuginone (HF) significantly reduced the number of iMEC colonies formed (Figures 4A,B). Then, we further applied RNA interference (RNAi) to down-regulate expression of *SMAD3*, and the results revealed that all three of the interfering target sequences could significantly decrease the expression level of *SMAD3* gene (Figure 4C). In order to determine whether reduced *SMAD3* gene expression affects RepSox-induced reprogramming of goat fibroblasts into mammary epithelial cells, we selected GEFs-shRNA2-SMAD3, which has the lowest *SMAD3* expression level. The results demonstrated that *SMAD3* downregulation severely affected RepSox-induced colonies formed (Figure 4D), and decrease the reprogramming efficiency to 0.85% from 3.56% (Figure 4E). The conclusion drawn from these findings is that *SMAD3* plays an important role in the conversion of fibroblasts into iMECs. Moreover, these findings provide further evidence that the RepSox-induced reprogramming of iMECs may be mediated through the regulation of SMAD3.

### SMAD3-iMECs have similar biological properties to R-CiMECs

**FIGURE 5:**
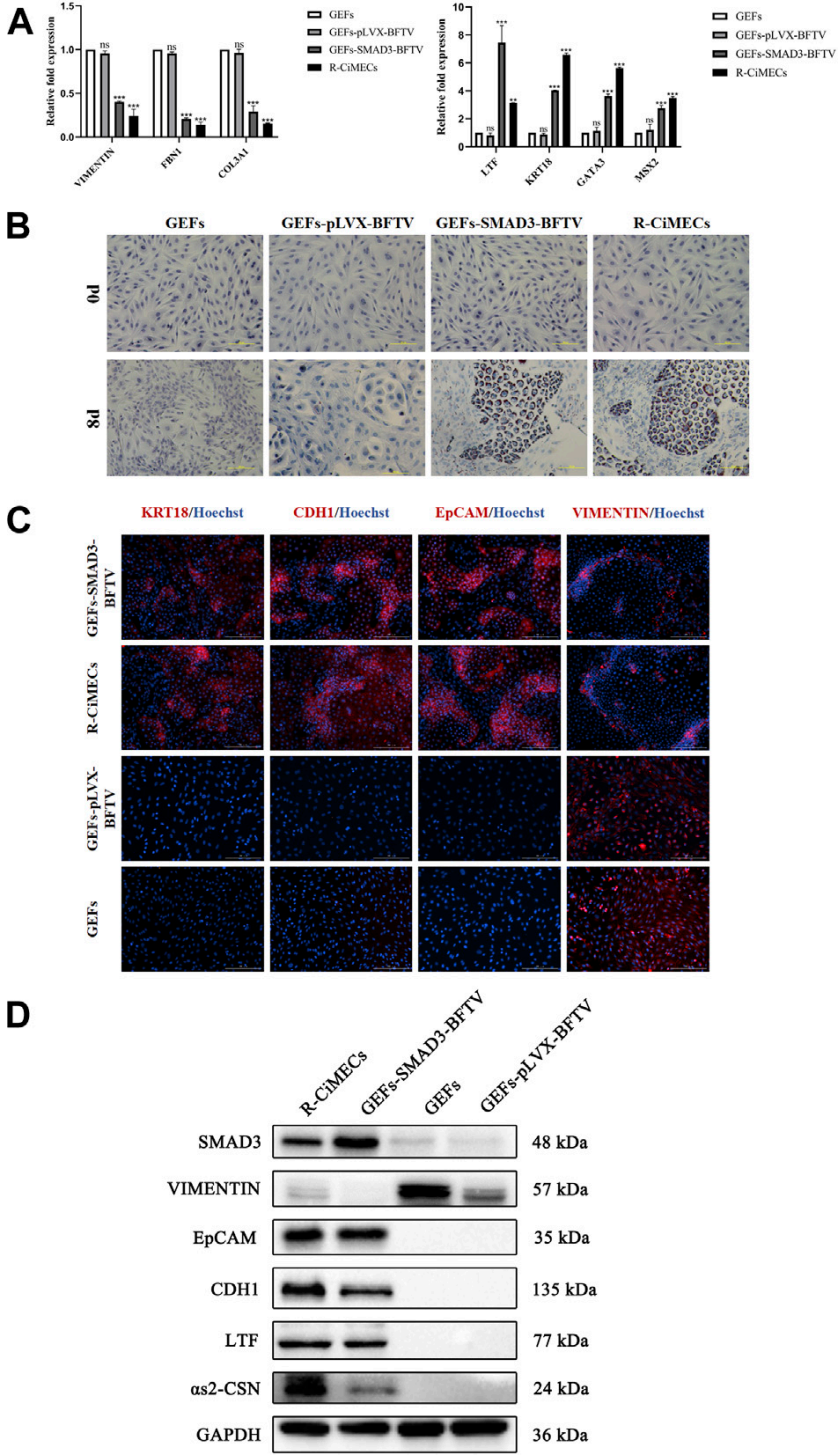
Biological characterization of SMAD3-iMECs. **(A)** RT-qPCR was performed to detect changes in the expression of fibroblast-related marker genes (*VIMENTIN*, *COL3A1*, and *FBN1*), MECs-related marker genes (*LTF* and *KRT18*), and MECs development-related genes (*MSX2* and *GATA3*) after induced mammary epithelial cell reprogramming in goats. *n* = 3 biological replicates. Data are represented as the mean ± SEM. **p < 0.01, ***p < 0.001 (one-way ANOVA). **(B)** Saturated oil red O staining was performed to identify SMAD3-iMECs with similar lipid droplet secretion functions as R-CiMECs. Scale bar, 100 μm. **(C)** Immunofluorescence staining was performed to detect the expression of fibroblasts marker antigen (VIMENTIN) and epithelial cell marker antigen (EpCAM, CDH1, and KRT18) in SMAD3-iMECs and R-CiMECs. Scale bar, 200 μm. **(D)** Western blot detection of SMAD3-iMECs and R-CiMECs with similar specific marker protein expression patterns (Fibroblasts marker protein: VIMENTIN; epithelial cell marker protein: CDH1 and EpCAM; mammary epithelial cell marker protein: LTF and αs2-CSN).

Finally, the biological characteristics of SMAD3-iMECs were identified. SMAD3-iMECs and R-CiMECs (positive control cells) strongly expressed the mammary epithelial cell marker genes *KRT18* and *LTF*, as well as the mammary development-related genes *MSX2* and *GATA3*, in comparison to the GEFs (negative control group cells). However, the fibroblast-related marker genes *FBN1*, *COL3A1,* and *VIMENTIN* were significantly downregulated (Figure 5A). An important feature of MECs is the ability to secrete milk fat. R-CiMECs and SMAD3-iMECs secreted lipid droplets of different sizes around the cytoplasm, which were stained red by saturated oil red O staining (Figure 5B), confirming their ability to secrete lipids. Furthermore, our immunofluorescence staining results showed that R-CiMECs and SMAD3-iMECs significantly expressed the epithelial cell-specific antigens CDH1, EpCAM, and KRT18, but not the fibroblasts-specific antigen VIMENTIN (Figure 5C). Western blot analysis revealed that SMAD3-iMECs expressed the lactating state mammary epithelial cell-specific proteins LTF and αs2-CSN, and the protein expression trend was similar to R-CiMECs (Figure 5D). These results indicated that SMAD3-iMECs with lactation function has similar biological properties to R-CiMECs.

## Discussion

Our previous report (Zhang et al., 2021) demonstrated that goat fibroblasts could be chemically induced to reprogram into mammary epithelial cells (CiMECs) by exposure to small molecule compounds (BFRTV). We further identified that RepSox (R), an inhibitor of TGFβR1, was the essential small molecule for this reprogramming, and revealed that R may act on CiMECs generation through the regulatory effects of SMAD3. In this study, we further proved that SMAD3 overexpression induced the conversion of goat fibroblasts into iMECs, and confirmed that SMAD3 plays a significant role in the iMECs reprogramming process. The regulatory mechanism of mammary epithelial cell fate may be better understood as a result of our discoveries.

The TGF-β/SMAD pathway is essential for the normal development of mammary epithelial tissue. Mammary epithelial cells exhibit significant levels of *Smad3* expression. In mice, *Smad3* deletion causes mammary development abnormalities secondary to ovarian insufficiency (Yang et al., 2002). Constitutive active Smad2/Smad3 is a broad-spectrum enhancer in the somatic reprogramming process regulated by transcription factors, which increases the efficiency of somatic reprogramming. Smad3 interacts with coactivators and reprogramming factors, combined with the OCT4 target motif, and significantly improves the efficiency of iPSCs generation by the Yamanaka factor (Ruetz et al., 2017). Smad2/Smad3 also significantly improved the efficiency of *CEBPα*-mediated transformation of B cells into macrophages (Ruetz et al., 2017). Additionally, Smad3 can directly reprogram spermatogonial stem cells into hepatocyte-like cells with mature hepatocyte morphology, phenotype, and function through the ERK1/2 and Smad2/3 signaling pathways (Zhang et al., 2013). Therefore, SMAD3 plays an important role in cellular reprogramming. Surprisingly, in this study, we not only demonstrated that *SMAD3* overexpression can initiate and complete the reprogramming of iMECs, but also that the reprogramming efficiency of iMECs shows a parabolic relationship with *SMAD3* expression levels. Moreover, the parabolic relationship might indicate that SMAD3 expression level is the key to achieving mammary epithelial cell fate in other species.

The primary cell type in the mammary gland is the mammary epithelial cells, which are crucial for lactation synthesis (Richert et al., 2000). Numerous signaling factors regulate the development and differentiation of mammary epithelial cells, among which Gata3 is a key transcription factor for luminal epithelial cell differentiation in the mammary gland (Kouros-Mehr et al., 2006; Siegel and Muller, 2010); targeted knockout of *Gata3* in the mammary gland results in severely impaired mammary gland development and inability to determine and maintain the fate of luminal epithelial cells (Asselin-Labat et al., 2007; Watson et al., 2011). In addition, Msx2 is a transcription factor necessary for mammary gland developmental differentiation, knockout of *Msx2* at E16.5 leads to a stagnation of mammary bud development, which is comparable to the mammary phenotype of conditional knockout *Gata3* mice (Phippard et al., 1996; Satokata et al., 2000). Our work discovered that *GATA3* and *MSX2* gene expression was upregulated in SMAD3-iMECs compared to fibroblasts, implying that *SMAD3* overexpression may activate the expression of mammary gland development-related genes such as *GATA3* and *MSX2*, which are involved in cell fate transformation. Last but not least, our findings showed that SMAD3-iMECs shared similar characterization with R-CiMECs, including MEC-specific markers and milk-secreting functions. Thus, these SMAD3-iMECs contained secretory luminal epithelial cells can secrete milk. It may suggest that SMAD3-iMECs have the capacity to generate mammary organoids for investigating mammary gland development and obtaining “culture dish milk” *in vitro*.

## Conclusion

In this study, we demonstrated that the regulatory role of SMAD3 is directly involved in mammary epithelial cell fate determination in goats, which offers novel insights into the regulatory mechanism of the TGFβR1-SMAD3 pathway. These findings may provide an alternative strategy for other species to regulate mammary epithelial cell fate *in vitro*. However, further experiments are still required to conduct in order to elucidate how SMAD3 expression level regulates mammary epithelial cell fate, and find downstream genes of SMAD3 on regulating reprogramming of iMECs.

## Data Availability

The original contributions presented in the study are included in the article/Supplementary Material, further inquiries can be directed to the corresponding author.
