# Supplementary material for: Parabolic relationship between SMAD3 expression level and the reprogramming efficiency of goat induced mammary epithelial cells
Source: Front Cell Dev Biol. 2022 Oct 14;10:1002874. doi: 10.3389/fcell.2022.1002874 (PMC9614088; doi:10.3389/fcell.2022.1002874)
Supplement: Supplementary file 1 [file Table1.DOCX]

**PCR amplification system of target gene**

| Component | Volume |
| --- | --- |
| Golden Star T6 Super PCR Mix | 45 μL |
| 10 μM forward primer | 2 μL |
| 10 μM reverse primer | 2 μL |
| 100 ng/μL Template DNA | 1 μL |

**PCR amplification protocol of target gene**

| Gene name | Step |  | Temperature | Time |
| --- | --- | --- | --- | --- |
| SMAD3 | Initial denaturation |  | 95 ℃ | 5min |
|  | -1 ℃/cycle 15 PCR cycles | Denature | 95 ℃ | 1 min |
|  |  | Anneal | 68 ℃ ⬇ | 15 sec |
|  |  | Extend | 72 ℃ | 2 min |
|  | 20 PCR cycles | Denature | 95 ℃ | 1 min |
|  |  | Anneal | 55 ℃ | 15 sec |
|  |  | Extend | 72 ℃ | 2 min |
|  | Final extension |  | 72 ℃ | 10 min |
|  |  |  | 4 ℃ | hold |
|  |  |  |  |  |
| P2A | Initial denaturation |  | 98 ℃ | 2 min |
|  | 30 PCR cycles | Denature | 98 ℃ | 10 sec |
|  |  | Anneal | 64 ℃ | 10 sec |
|  |  | Extend | 72 ℃ | 15 sec |
|  | Final extension |  | 72 ℃ | 5 min |
|  |  |  | 4 ℃ | hold |
|  |  |  |  |  |
| ZsGreen1 | Initial denaturation |  | 98 ℃ | 2 min |
|  | 30 PCR cycles | Denature | 98 ℃ | 10 sec |
|  |  | Anneal | 62 ℃ | 10 sec |
|  |  | Extend | 72 ℃ | 15 sec |
|  | Final extension |  | 72 ℃ | 5 min |
|  |  |  | 4 ℃ | hold |

**SOE PCR system 1**

| Component | Volume |
| --- | --- |
| Water, nuclease-free | to 23 μL |
| 2×Phanta Flash Master Mix（Dye Plus） | 12.5 μL |
| DNA a（SMAD3） | 1.25 ng |
| DNA b（P2A） | 1.25 ng |

**SOE PCR protocol 1**

| Step |  | Temperature | Time |
| --- | --- | --- | --- |
| Initial denaturation |  | 98 ℃ | 30 sec |
| 5 PCR cycles | Denature | 98 ℃ | 10 sec |
|  | Anneal | 64 ℃ | 5 sec |
|  | Extend | 72 ℃ | 8 sec |
| Final extension |  | 72 ℃ | 1 min |
|  |  | 4 ℃ | 10 min |

**SOE PCR system 2**

| Component | Volume |
| --- | --- |
| 10 μM forward primer for a | 1 μL |
| 10 μM reverse primer for b | 1 μL |
| Reagent from SOE PCR setup 1 | 23 μL |

**SOE PCR protocol 2**

| Step |  | Temperature | Time |
| --- | --- | --- | --- |
| Initial denaturation |  | 98 ℃ | 30 sec |
| 30 PCR cycles | Denature | 98 ℃ | 10 sec |
|  | Anneal | 64 ℃ | 5 sec |
|  | Extend | 72 ℃ | 8 sec |
| Final extension |  | 72 ℃ | 1 min |
|  |  | 4 ℃ | hold |

**Reaction system of plasmid double enzyme digestion**

| Component | Volume |
| --- | --- |
| Water, nuclease-free | to 50 μL |
| Plasmid | 5 μg |
| 10× CutSmart Buffer A | 5 μL |
| 10× CutSmart Buffer B | 5 μL |
| Restriction endonuclease A | 2 μL |
| Restriction endonuclease B | 2 μL |

**Reaction system of plasmid double enzyme digestion**

| Temperature | Time |
| --- | --- |
| 37 ℃ | 4 h |
| 4 ℃ | hold |

**Reaction system of homologous recombination**

| Component | Volume |
| --- | --- |
| Water, nuclease-free | to 10 μL |
| 5× CE MultiS Buffer | 2 μL |
| Exnase MultiS | 1 μL |
| Purified PCR fragments | 42 ng |
| Linearized vector | 184 ng |

**Reaction system of homologous recombination**

| Temperature | Time |
| --- | --- |
| 37 ℃ | 30 min |
| 4 ℃ | hold |

**PCR reaction system of bacterial solution**

| Component | Volume |
| --- | --- |
| Water, nuclease-free | to 20 μL |
| 2× Rapid Taq Master Mix | 10 μL |
| 10 μM forward primer | 1 μL |
| 10 μM reverse primer | 1 μL |
| Bacterium solution | 2 μL |

**PCR reaction procedure of bacterial solution**

| Step |  | Temperature | Time |
| --- | --- | --- | --- |
| Initial denaturation |  | 95 ℃ | 10 min |
| 30 PCR cycles | Denature | 95 ℃ | 15 sec |
|  | Anneal | Tm | 15 sec |
|  | Extend | 72 ℃ | 15 sec/kb |
| Final extension |  | 72 ℃ | 5 min |
|  |  | 4 ℃ | hold |

**Reaction system of shRNA annealing**

| Component | Volume |
| --- | --- |
| 100 μM forward primer for shRNA | 4.5 μL |
| 100 μM reverse primer for shRNA | 4.5 μL |
| 10× LA Buffer | 1 μL |

**Reaction system of shRNA annealing**

| Temperature | Time |
| --- | --- |
| 95 ℃ | 10 min |
| 25 ℃ | 30 min |
| 4 ℃ | hold |

**Reaction system of T4 DNA ligation**

| Component | Volume |
| --- | --- |
| Water, nuclease-free | to 20 μL |
| Linearized vector | 100 ng |
| Double stranded shRNA | 500-1000 ng |
| 10× T4 DNA Buffer | 2 μL |
| T4 DNA Ligase | 1 μL |
| 50% PEG 4000 solution | 2 μL |

**Reaction system of T4 DNA ligation**

| Temperature | Time |
| --- | --- |
| 16 ℃ | 5 h |
| 4 ℃ | hold |

**Antibody source and dilution ratio**

| **REAGENT or RESOURCE** | **SOURCE** | **IDENTIFIER** | **Dilution** |
| --- | --- | --- | --- |
| **First Antibodies** | | |  |
| Rabbit E-Cadherin (24E10) (CDH1) for IF/WB | CST | Cat# 3195;  RRID: AB_2291471 | IF: 1: 200  WB: 1: 1000 |
| Mouse monoclonal anti-Cytokeratin 18 (KRT18) for IF | Novus | Cat# NBP2-44951; RRID: AB_2811032 | 1: 100 |
| Rabbit polyclonal anti-EPCAM (EPCAM) for IF/WB | Abcam | Cat# ab71916;  RRID: AB_1603782 | IF: 1: 100  WB: 1: 1000 |
| Rabbit polyclonal anti-Lactoferrin (LTF) for WB | LSBio | Cat# LS-C295067;  RRID: AB_2811036 | 1: 1000 |
| Mouse monoclonal anti-Vimentin (VIM) for IF/WB | Santa | Cat# sc-32322;  RRID: AB_628436 | IF: 1: 100  WB: 1: 1000 |
| Rabbit Anti-Alpha s2 Casein antibody (αs2-CSN) for WB | Bioss | Cat# bs-10034R;  RRID: AB_2884955 | 1: 1000 |
| Rabbit monoclonal [EP568Y] to Smad3 for WB | Abcam | Cat# ab40854;  RRID: AB_777979 | 1: 1000 |
| GAPDH mouse monoclonal antibody for WB | Proteintech | Cat# HRP-60004;  RRID: AB_2737588 | 1: 5000 |
| **Second Antibodies** | | |  |
| HRP-conjugated Affinipure Goat Anti-Rabbit IgG (H+L) for WB | Proteintech | Cat# SA00001-2;  RRID: AB_2722564 | 1: 10000 |
| HRP Goat Anti-Mouse IgG (H+L) for WB | ABclonal | Cat# AS003;  RRID: AB_2769851 | 1: 10000 |
| Alexa Fluor 555 Donkey Anti-rabbit for IF | Abcam | Cat# ab150074;  RRID: AB_2636997 | 1: 250 |
| Alexa Fluor 594 Donkey Anti-mouse for IF | Abcam | Cat# ab150108;  RRID: AB_2732073 | 1: 250 |
